# Ovarian Microvascular Endothelial Cell Migration Is Dysregulated in PMOS

**DOI:** 10.3390/cimb48100979

**Published:** 2026-09-24

**Authors:** Arielle S. Yeshua, Megan A. G. Sage, Diane M. Duffy

**Affiliations:** 1 The Jones Institute, Eastern Virginia Medical School, Old Dominion University, Norfolk, VA 23507, USA; 2 Department of Obstetrics and Gynecology, Eastern Virginia Medical School, Old Dominion University, Norfolk, VA 23507, USA; 3 Department of Biomedical and Translational Sciences, Eastern Virginia Medical School, Old Dominion University, Norfolk, VA 23507, USA; sagemag@odu.edu (M.A.G.S.); duffydm@odu.edu (D.M.D.)

**Keywords:** polyendocrine metabolic ovarian syndrome, polycystic ovary syndrome, angiogenesis, microvasculature, prostaglandin E2, VEGFR1, VEGFR2, PTGER

## Abstract

The objective of this study is to investigate whether ovarian microvascular endothelial cells (OMECs) from women with polyendocrine metabolic ovarian syndrome (PMOMECs) exhibit altered angiogenic responses compared with cells from healthy oocyte donors (HOMECs). This is a laboratory-based cross-sectional study using HOMECs and PMOMECs obtained from discarded follicular aspirates. Expression of mRNA and protein for VEGF receptors (FLT1, KDR) and PGE2 receptors (PTGER1–4) were assessed using qPCR and immunofluorescence. OMECs were treated in vitro with VEGFA, PGE2, or receptor-selective agonists. Migration was quantified via porous membrane inserts and live cell tracking. Proliferation was measured via BrdU incorporation. FLT1 and KDR mRNA and protein levels were similar between HOMECs and PMOMECs. PTGER2 mRNA was higher and PTGER3 mRNA was lower in PMOMECs. VEGFA and PGE2 significantly stimulated migration and proliferation in HOMECs. In contrast, PMOMECs failed to exhibit increased migration in response to PGE2 or selective PTGER agonists, while VEGFA-induced migration remained comparable to migration of HOMECs. Live cell tracking revealed that PMOMEC migration resulted in decreased overall net distance traveled and reduced directional persistence (less linear movement) compared with HOMECs. These findings demonstrate that ovarian microvascular endothelial cells from women with PMOS display impaired angiogenic responses to PGE2, which may contribute to altered follicle growth and anovulation in PMOS.

## 1. Introduction

Polyendocrine metabolic ovarian syndrome (PMOS) is the most common endocrine disorder among women. Previously known as polycystic ovary syndrome (PCOS), PMOS affects an estimated 6–12% of women in the US and worldwide [1]. PMOS is characterized by infrequent or absent ovulation, polycystic ovarian morphology, and clinical or biochemical evidence of hyperandrogenism [2]. Current therapies primarily target symptoms, such as hirsutism and infertility, as the underlying causes of PMOS remain poorly understood.

Women with PMOS are at increased risk of cardiovascular disease and pregnancy-related cardiovascular complications, even after accounting for associated factors such as obesity and insulin resistance [3,4]. These risks may reflect underlying macrovascular and microvascular differences between women with and without PMOS [3,5]. The ovarian vasculature is altered in women with PMOS [5]. Color Doppler studies demonstrate increased ovarian stromal blood flow and vascularity in women with PMOS compared with controls [6,7]. Notably, distinct interventions, including metformin [8,9] and, historically, laparoscopic ovarian drilling [10,11], have been shown to normalize ovarian stromal blood flow and improve ovulatory function in women with PMOS, indirectly suggesting a link between the ovarian vasculature and healthy ovarian follicles.

Abnormal ovarian follicle development is a hallmark of PMOS. Ovaries in PMOS often accumulate large numbers of antral follicles (2–8 mm in diameter), yet many of these follicles fail to progress to the typical preovulatory size (16–22 mm) and ovulate [2]. The ovarian microvasculature is a key regulator of follicle growth and maturation. Primordial and primary follicles do not have a dedicated vascular supply [12]. Vascularization is essential as follicles transition to the secondary and antral stages [12,13,14]. Follicles with a richer vascular supply show improved growth and a greater likelihood of progressing to the antral stage and beyond [15,16]. Healthy antral follicles exhibit a greater density of capillaries within the theca layer [17,18]. As follicles mature, pro-angiogenic factors such as vascular endothelial growth factor A (VEGFA) and prostaglandin E2 (PGE2) play pivotal roles in promoting endothelial cell migration, proliferation, and capillary sprouting [12]. In PMOS, altered follicular vasculature may contribute to abnormal follicle growth and resulting hyperandrogenism [17,19]. Follicular fluid and serum levels of vascular regulators, such as VEGFA and PGE2, are elevated in women with PMOS [13,14,20]. Our prior studies demonstrate that VEGFA and PGE2 stimulate ovarian microvascular endothelial cells (OMECs) from healthy oocyte donors (HOMECs) to migrate and proliferate in vitro [21]. However, the function of ovarian endothelial cells in women with PMOS has not been examined.

To address this gap, we investigated whether ovarian microvascular endothelial cells healthy oocyte donors (HOMECs) and women with PMOS (PMOMECs) exhibit differential angiogenic responses. We hypothesized that PMOMECs would show impaired angiogenic activity compared to HOMECs. Using in vitro treatment with PGE2, VEGFA, and selective receptor agonists, we aimed to determine whether PMOS is associated with altered microvascular endothelial cell responses that could contribute to abnormal follicle development.

## 2. Materials and Methods

### 2.1. Human Ovarian Microvascular Endothelial Cells

Ovarian microvascular endothelial cells (OMECs) were isolated from discarded human follicular aspirates obtained during oocyte retrieval at the Jones Institute for Reproductive Medicine, Eastern Virginia Medical School (EVMS) at Old Dominion University, Norfolk, VA, USA. Primary cell lines were established from two distinct patient cohorts: healthy women undergoing ovarian stimulation for oocyte donation (HOMECs) and patients diagnosed with PMOS undergoing ovarian stimulation for in vitro fertilization (PMOMECs). Inclusion criteria for donor controls included required age 19–31 years, regular menstrual cycles (21–35 days), normal baseline serum hormone levels (including anti-Müllerian hormone [AMH], basal FSH, and androgens), body mass index 18 to 29 kg/m^2^, normal baseline pelvic ultrasonography showing no features of polycystic ovarian morphology, non-smoker, and no personal or family history of metabolic or reproductive disorders. Patients in the PMOS group met the Rotterdam consensus criteria for PMOS diagnosis, requiring at least two of the following three features: oligo- or anovulation; clinical and/or biochemical hyperandrogenism; polycystic ovarian morphology on pelvic ultrasound (≥12 antral follicles measuring 2–9 mm per ovary or increased ovarian volume ≥ 10 cm^3^ [2]). Exclusion criteria for all participants included secondary causes of hyperandrogenism (such as congenital adrenal hyperplasia, Cushing’s syndrome, or androgen-secreting tumors). This use of discarded human aspirates does not constitute human subjects research as determined by the EVMS Institutional Review Board. The EVMS Institutional Review Board did permit identifying aspirates from oocyte donors and PMOS patients without additional patient information. While no specific treatment data are available, follicular aspirates are routinely obtained about 36 h after administration of an ovulatory trigger. After oocyte removal, remaining aspirated cells were plated in fibronectin-coated flasks in Endothelial Growth Medium-2 (EGM2) media (Lonza, Walkersville, MD, USA), which is optimized for culture of microvascular endothelial cells. Once cells reached confluence (a tightly packed monolayer covering 90–100% of the culture dish), endothelial cells were isolated using CD31 Dynabeads (Invitrogen, Carlsbad, CA, USA) following the manufacturer’s protocol [22,23]. A total of 4 HOMEC and 6 PMOMEC lines were established, each from an individual woman.

Primary replicating populations of HOMECs and PMOMECs were characterized as >95% microvascular endothelial cells (Figure S1). Immunocytochemistry confirmed detection of the endothelial cell protein von Willebrand factor (VWF) [24], with limited detection of smooth muscle actin (SMA) [25] or the granulosa cell protein aromatase (CYP19A1) [26] as previously reported [21,22]. HOMECs and PMOMECs also demonstrated uptake of acetylated low-density lipoprotein (Alexa Fluor 488 AcLDL; Invitrogen [27]) and binding of the lectin ulex europaeus agglutinin I (FITC-UEA-1; Invitrogen [28]) as previously reported [21,22] (Figure S1). In preliminary studies, OMECs maintained their phenotype through 10 passages. In this paper, we report data from passages 4–7. OMECS were maintained on fibronectin-coated culture ware in EGM2 medium (Lonza). Media was changed to Endothelial Basal Medium-2 (EBM2) (Lonza) + 1% fetal bovine serum (Gibco, Grand Island, NY, USA) overnight before use in migration and proliferation assays, which were conducted in serum-free EBM2 media (basal).

### 2.2. RNA Isolation, Amplification, and Quantitative PCR

HOMECs and PMOMECs were grown to confluence. RNA was extracted using the RNeasy^®^ Mini Kit (Qiagen, Germantown, MD, USA) per manufacturer’s instructions. Quality and quantity of RNA was assessed via NanoDrop 1000 (NanoDrop Technologies, Wilmington, DE, USA). RNA (400 ng) was converted to cDNA using the Qiagen RT2 First Strand Kit (Germantown, MD, USA) per manufacturer’s instructions. All primers were designed based on human sequences and span an intron to prevent undetected amplification of genomic DNA (Table S1). Quantitative PCR (qPCR) was performed using the FastStart SYBR^®^ Green Master kit (Roche Diagnostics GmbH; Mannheim, Germany) per manufacturer’s instructions with the CFX96 Real-Time System (Bio-Rad Laboratories, Hercules, CA, USA). Cycling conditions were as follows: 10 min at 95 °C; then amplification cycles of 15 s at 95 °C, 30 s to anneal (Table S1), and 50 s at 72 °C. A melt curve was performed ranging from 65 °C to 95 °C in 0.5 °C increments. Expression of target mRNA was calculated via 2^−ΔΔCT^ method and normalized to expression of GAPDH.

### 2.3. Immunocytochemistry

HOMECs and PMOMECs were grown to confluence on chamber slides, then fixed as previously described [22] and used for immunodetection of VEGF and PGE2 receptors. Prior to immunodetection of FLT1 and KDR, OMECs were exposed to basic antigen retrieval as previously described [29]. After blocking, OMECs were incubated with an antibody directed against a single PGE2 receptor (PTGER1 (1:300, Cayman Chemical Cat# 101740, Ann Arbor, MI, USA, RRID:AB_10079426), PTGER2 (1:300, Cayman Cat# 101750, RRID:AB_10078697), PTGER3 (1:200, Cayman Cat# 101760-1, RRID:AB_327849), PTGER4 (1:200, Cayman Cat# 101770-1, RRID:AB_327978)) or a single VEGF receptor (FLT1 (0.67 µg/mL, Santa Cruz Biotechnology Cat# sc-316, Santa Cruz, CA, USA, RRID:AB_2107031), KDR (1.33 µg/mL, Santa Cruz Biotechnology Cat# sc-505, RRID:AB_632601)). Omission of the primary antibody served as a negative control. OMECs were then incubated with Alexa Fluor 488-conjugated anti-rabbit secondary antibody (2 µg/mL; ThermoFisher, Waltham, MA, USA), followed by 1% Sudan Black (in 70% methanol) and 4′,6-diamidino-2-phenylindole (DAPI) counterstain (ProLong Gold Antifade Mountant, Invitrogen). Images were acquired using the Keyence BZ-X Analyzer (Keyence Corp., Osaka, Japan).

### 2.4. Proliferation Assay

Proliferation was quantified by 5-bromo-2′-deoxyuridine (BrdU) incorporation. OMECs were grown to 50% confluence on chamber slides. OMECs were cultured for 24 h in EBM2 medium alone (basal) or with the addition of VEGFA (5 ng/mL, R&D Systems, Minneapolis, MN, USA), VEGFE (5 ng/mL, Fitzgerald Industries International, Acton, MA, USA), PGF (5 ng/mL, R&D Systems), PGE2 (10^−6^ M or 10^−8^ M, Cayman), 17-phenyl-trinor-PGE2 (17PTP; 10^−6^ M, Cayman), butaprost (But; 10^−5^ M, Cayman), sulprostone (Sul; 10^−6^ M, Cayman), or PGE1 alcohol (PGE1OH; 10^−6^ M, Cayman) [21]. VEGFA is a well-recognized key regulator of angiogenesis within the ovulatory follicle [30,31,32]. PGF and VEGFE were used as selective agonists for the VEGFA receptors FLT1 and KDR, respectively [21]. HOMEC and PMOMEC responses to PGE2 were evaluated at concentrations consistent with high receptor occupancy and ovulatory follicular fluid levels [33,34,35,36]. 17PTP, butaprost, sulprostone, and PGE1 alcohol were utilized as agonists selective for PTGER1, PTGER2, PTGER3, and PTGER4 respectively [35]. Vehicles (<0.01% of ethanol or dimethyl sulfoxide (DMSO)) did not alter OMEC proliferation or migration in prior studies [21]. BrdU (50 µM) was added to each well 4 h prior to cell fixation, based on preliminary experiments testing multiple BrdU concentrations (10–200 µM) and labeling durations (4–8 h). BrdU immunodetection proceeded according to kit instructions (Invitrogen Cat #8841659945). Images (9 per well) were obtained in a standard, unbiased pattern with the Keyence BZ-X Analyzer (Keyence Corp., Osaka, Japan). BrdU-positive and BrdU-negative nuclei were quantified using Keyence BZ-X Analyzer software version 1.1.30.19.

### 2.5. Migration Assay

Migration of HOMECs and PMOMECs was assessed using 8 μm pore membrane inserts (BD Biosciences, San Jose, CA, USA) as previously described [21]. OMECs (150,000 cells per membrane) were seeded in the upper chamber, and the lower chamber contained EMB2 media alone (basal) or with agonists as described for the proliferation assay. Vehicles (<0.01% of ethanol or DMSO) did not alter OMEC migration in prior studies [21]. After 24 h at 37 °C, non-migrated cells were removed from the upper membrane surface. Migrated cells (lower membrane surface) were fixed in 70% ethanol for 2 min and stained with hematoxylin and eosin. Membranes were air-dried overnight, and five fields per membrane were imaged in a standard, unbiased pattern to quantify migrated cells.

### 2.6. Live Cell Tracking for Migration

Migration of HOMECs and PMOMECs was assessed using live cell tracking. OMECs (1000 cells per well) were seeded onto a glass coverslip-bottom 96 well plate containing EMB2 media without or with VEGFA (5 ng/mL) or PGE2 (10^−6^ M). Cells were incubated in a humidified chamber at 37 °C with 5% CO_2_, and wells were imaged every 20 min for 16 h using phase contrast microscopy via the BZ-X1000 fluorescence microscope (Keyence Corp.). Thirty cells were analyzed for each cell line, for a total of 120 HOMECs (*n* = 4 lines) and 150 PMOMECs (*n* = 5 lines). The change in x and y position (microns) of each cell was determined for every 20 min imaging interval. This positional data was used to generate spaghetti plots and additional trajectory analysis, including distance traveled (path length), total displacement, speed, and persistence using the dynamic tracking module of the BZ-X Analyzer (Keyence Corp.).

### 2.7. Data Analysis

Data were assessed for heterogeneity of variance by Bartlett’s test. Data were log transformed when Bartlett’s test yielded *p* < 0.05; log-transformed data were subjected to Bartlett’s test to confirm that *p* > 0.05. As indicated in the figure legends, data sets were assessed by 2-tail paired *t*-test or ANOVA (without or with repeated measures) following by Duncan’s multiple range test (StatPak version 4.12 software; Northwest Analytical, Portland, OR, USA). Live cell tracking data were log-transformed and analyzed in linear mixed effects models with primary cell line as a cluster variable and post hoc comparisons with Holm correction using jamovi software version 2.6.45.0 jamovi, (Sydney, Australia). Significance was assumed at *p* < 0.05. Data are expressed as mean + SEM.

## 3. Results

### 3.1. VEGFA and PGE2 Receptor Expression in HOMECs and PMOMECs

VEGFA can act via multiple receptors (Figure 1A). HOMECs and PMOMECs expressed mRNA for the VEGFA receptors FLT1 (also known as VEGFR1; Figure 2A) and KDR (also known as VEGFR2; Figure 2B). HOMECs and PMOMECs had similar levels of FLT1 mRNA and KDR mRNA. Immunodetection confirmed expression of FLT1 and KDR proteins in both HOMECs and PMOMECs (Figure 2G–J).

PGE2 also acts via multiple receptors (Figure 1B). HOMECs and PMOMECs expressed mRNA for PTGER1, PTGER2, PTGER3, and PTGER4 (Figure 2C–F). PMOMECs had significantly higher levels of PTGER2 mRNA (Figure 2D) and significantly lower levels of PTGER3 mRNA (Figure 2E) when compared with HOMECS. Similar levels of PTGER1 mRNA and PTGER4 mRNA were found in HOMECs and PMOMECs (Figure 2C,F).

PTGER proteins were also present in HOMECs and PMOMECs. PTGER1, PTGER2, and PTGER4 were present throughout HOMECs and PMOMECs, consistent with the anticipated plasma membrane location of these receptor proteins (Figure 2K–N,Q,R). PTGER3 was detected throughout HOMECs and PMOMECs, with concentration noted near the nucleus (Figure 2O,P). Perinuclear localization was most notable in PMOMECs (Figure 2P).

### 3.2. VEGF Receptor Agonists and PGE2 Promote Endothelial Cell Proliferation in HOMECs and PMOMECs

Proliferation is a key angiogenic response to extend the length of newly forming capillaries. HOMECs and PMOMECs proliferated in response to VEGF receptor agonists. VEGFA treatment increased proliferation above basal levels in HOMECs (Figure 3A) and PMOMECs (Figure 3C). PGF and VEGFE were utilized as agonists selective for FLT1 and KDR, respectively (Figure 1A). The FLT1 agonist PGF increased proliferation in both HOMECs (Figure 3A) and PMOMECs (Figure 3C). In contrast, the KDR agonist VEGFE did not alter proliferation in either HOMECs or PMOMECs (Figure 3A,C).

### 3.3. VEGFA Promotes Endothelial Cell Migration in HOMECs and PMOMECs

Migration is essential for initiating formation of new capillaries. VEGFA stimulated robust migration above basal levels in both HOMECs (Figure 4A) and PMOMECs (Figure 4D). PGF and VEGFE also increased migration above basal levels in both HOMECs and PMOMECs (Figure 4A,D).

### 3.4. PGE2 Promotes Endothelial Cell Migration in HOMECs but Not PMOMECs

PGE2 significantly stimulated HOMEC migration above basal levels at the higher concentration tested (Figure 4B). In contrast, PGE2 did not increase migration in PMOMECs above basal levels at either concentration tested (Figure 4E).

### 3.5. PMOMECs Migrate with Reduced Distance and Persistence When Compared to HOMECs

Live cell tracking was used to analyze the migratory trajectories of individual HOMECs and PMOMECs over time (Videos S1 and S2). HOMECs (Figure 4M) traveled farther from their starting position than did PMOMECs (Figure 4N). Displacement, the distance between the starting and ending position of the cell, was significantly greater for HOMECs than for PMOMECs (Figure 4O). HOMECs also demonstrated greater persistence than PMOMECs, which is the ratio of displacement to total distance traveled by the cell in its trajectory, with greater ratios being indicative of cells moving consistently in one direction (Figure 4P). Distance traveled (Figure 4Q) and speed (Figure 4R) were not different between HOMECs and PMOMECs.

## 4. Discussion

In this study, we demonstrate that ovarian microvascular endothelial cells from women with PMOS exhibit impaired angiogenic responses to PGE2 when compared with endothelial cells from healthy oocyte donors. Specifically, PGE2stimulated migration in OMECs from healthy ovulatory women, but OMECs from women with PMOS failed to respond to PGE2 with enhanced migration, a deficit recapitulated by selective agonists for each PGE2 receptor. PMOMECs had lower overall migratory displacement and persistence than HOMECs independent of treatment. These findings provide novel evidence that microvascular endothelial dysfunction in PMOS selectively affects prostaglandin-mediated vascular remodeling, which may contribute to abnormal follicle growth and ovulation failure in women with PMOS.

Migration responses were impaired in PMOMECs. PGE2, regardless of concentration, failed to enhance migration in PMOMECs. Receptor-selective PTGER agonists reproduced this pattern. PGE2 and each PGE2 receptor-selective agonist increased migration in HOMECs, consistent with prior studies in human and monkey ovarian microvascular endothelial cells [21,22], whereas no PGE2 receptor stimulus enhanced migration in PMOMECs. Importantly, VEGFA and VEGFA receptor-selective agonists enhanced migration in both HOMECs and PMOMECs. These findings demonstrate that PMOMECs are functionally competent to migrate in response to select angiogenic stimuli. The impairment of PGE2-mediated migration in PMOMECs suggests a specific alteration in prostaglandin signaling within the ovarian microvasculature in PMOS. This is consistent with our receptor expression data, showing elevated PTGER2 and reduced PTGER3 mRNA in PMOMECs, potentially altering the balance of downstream signaling pathways that promote endothelial cell migration. In studies of vascular endothelial cells from organs as diverse as brain, retina, and placenta, PGE2 receptors and post-receptor signaling have been linked to altered endothelial cell migration, microvascular anomalies, and disease progression [39,40,41,42]. Together, these findings support the concept that aberrant PGE2 signaling may selectively compromise endothelial cell migration in PMOS.

Live cell tracking provides additional mechanistic insight into the migratory phenotype of PMOMECs. PMOMECs exhibited reduced net displacement and persistence, despite unchanged migration speed and distance traveled, when compared to HOMECs. These findings indicate that PMOMECs retain motile capacity but display more randomly oriented migration. Persistent, directional migration is essential for angiogenesis, as endothelial cells must migrate using angiogenic gradients or extracellular matrix cues to form organized vascular networks [43,44]. Disruption of mechanisms that promote persistent, directional migration has been found to impair angiogenesis despite preserved motility, indicating that efficient vascular sprouting may require organized directional movement regardless of migratory speed or distance traveled [45]. Abnormal ovarian angiogenesis has been reported in PMOS [14]. Impaired directional migration could disrupt microvascular remodeling, contributing to inadequate vascular support for follicle growth and maturation in PMOS.

PGE2 stimulated proliferation in both HOMECs and PMOMECs, indicating that signaling via PGE2 receptors is preserved in OMECs from women with PMOS. VEGFA also promoted proliferation in both HOMECs and PMOMECs. Using receptor-selective agonists, we confirmed that FLT1 activation increased proliferation, whereas KDR activation had no effect. These results are consistent with our prior studies showing that stimulation of FLT1, but not KDR, promotes proliferation in HOMECs [21]. They also align with a broader body of evidence indicating that FLT1 drives proliferation in endothelial cells, whereas KDR predominantly mediates migration and capillary sprouting [37,46,47,48]. Importantly, our findings demonstrate that PMOMECs retain the capacity to respond to both PGE2 and VEGFA. Women with PMOS show evidence of microvascular dysfunction in tests of flow-mediated dilation [49] as well as altered responses to insulin, acetylcholine, and other stimuli [50,51,52,53]. Furthermore, recent disease-modeling using PMOS-specific iPSC-derived ECs has provided direct evidence of both endogenous and androgen-mediated endothelial dysfunction, revealing that PMOS endothelial cells possess intrinsic molecular impairments and a blunted angiogenic responses [54]. Our studies align with this growing body of work showing that endothelial dysfunction is a feature of PMOS, with altered vascular responses limited to select organs, functions, and signaling pathways.

Primordial and primary follicles lack a dedicated vasculature, relying on diffusion for gas exchange and supply of nutrients (reviewed in [12,15]). As follicles transition from the secondary to antral stage, vascularization of the theca layer supports continued growth [12,13,15]. The healthiest antral follicles exhibit a greater density of capillaries within the theca layer, supporting continued growth and counteracting hypoxia that develops as the follicle enlarges [15]. Immediately after the ovulatory gonadotropin stimulus, rapid angiogenesis within the luteinizing granulosa cell layer supports development of a functional corpus luteum (reviewed in [55]). During these later stages of follicle growth and into luteal development, intraovarian angiogenic mediators including VEGFA and PGE2 are key to vascular remodeling and new capillary formation [12,55].

In PMOS, follicle growth often stalls at the small antral stage [17,18], despite increased overall ovarian vascularity [13,14,17,56]. The cause of antral follicle growth arrest in PMOS remains unknown and is likely multifactorial, with proposed mechanisms including premature granulosa cell differentiation and inadequate FSH support [57,58,59]. Increasingly, studies highlight qualitative defects in ovarian vascular function as contributors to reproductive dysfunction in PMOS [19]. Clinical evidence suggests that up-regulation of angiogenic factors, both in serum and within the ovary, may contribute to the dysregulation of early folliculogenesis in women with PMOS [60,61,62,63]. Many studies confirm elevated levels of key angiogenic factors, including VEGFA, PGF, and PGE2, in follicular fluid of women with PMOS at oocyte retrieval [64,65]. A recent study by Patil and colleagues demonstrated that angiogenesis-related genes and their microRNA regulators are dysregulated in ovarian cells from women with PMOS [63], suggesting an additional mechanism may impair ovarian vascularization and follicular development. Adding to this evidence, our findings indicate that endothelial cell dysfunction, specifically impaired PGE2-mediated migration, may disrupt the dynamic microvascular remodeling required to support healthy antral follicle growth and ovulatory angiogenesis.

Strengths of this study include the use of primary human OMECs from well-characterized PMOS patients and healthy oocyte donors, functional assessment of OMEC proliferation and migration, and evaluation of receptor-specific responses. Limitations include a modest sample size and the in vitro nature of the assays, which may not fully reflect in vivo vascular dynamics. While these studies assessed receptor expression and functional outcomes, post-receptor signaling pathways were not evaluated. Future investigations should explore the molecular mechanisms underlying impaired PGE2-mediated signaling in PMOMECs, including downstream effectors of PTGERs and potential interactions with other angiogenic factors. Translational studies examining whether modulation of prostaglandin signaling can improve the ovarian microvasculature and better support follicle growth in PMOS may hold clinical relevance.

In conclusion, our findings indicate that ovarian microvascular endothelial cells from women with PMOS display a reduced overall migratory phenotype, with selective impairment in PGE2-mediated angiogenic responses. Reduced endothelial migration may contribute to disrupted follicle growth and anovulation observed in PMOS. Our results highlight the importance of the ovarian microvasculature in follicular maturation and provide a framework for future studies examining endothelial cell dysfunction in the pathophysiology of PMOS.

## Figures and Tables

**Figure 1:**
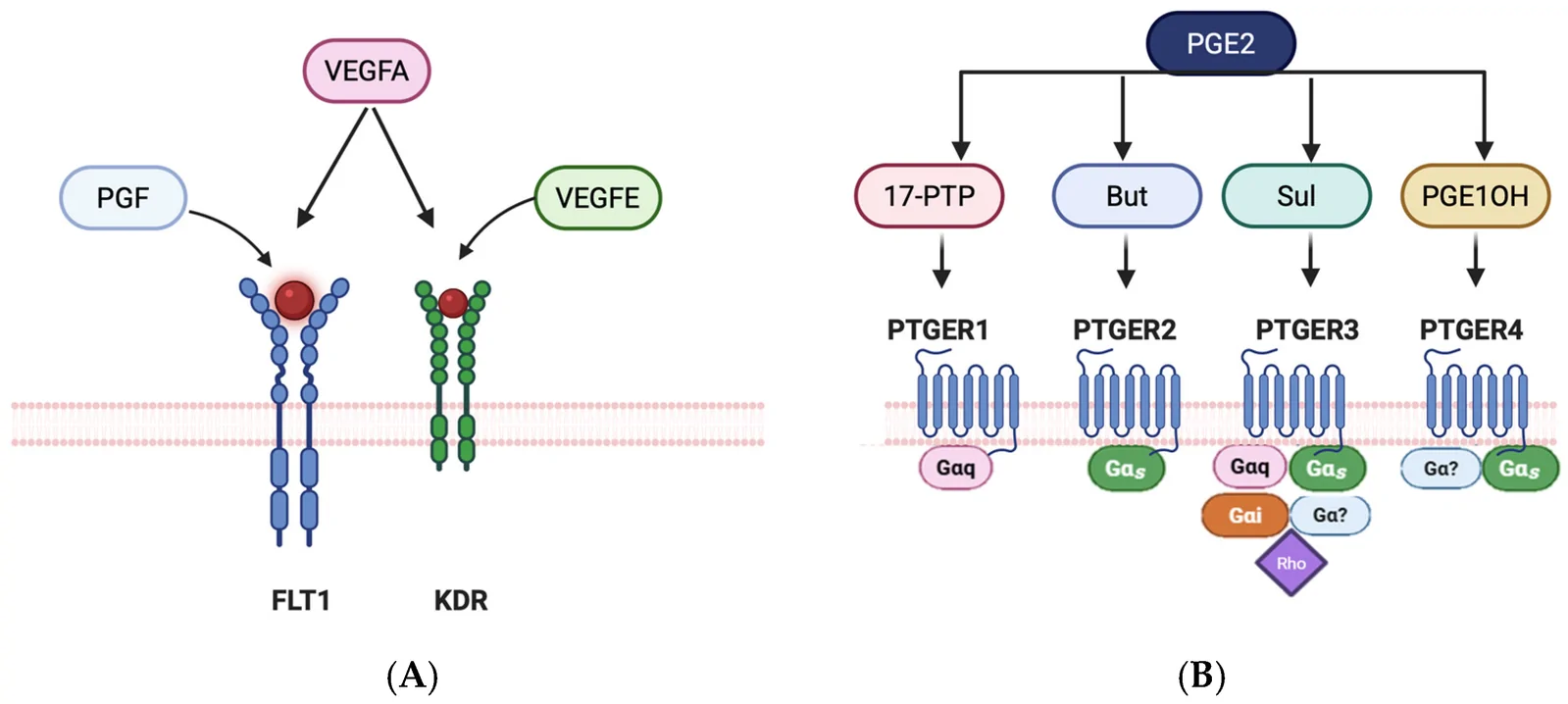
Receptors and selective agonists. Panel (**A**). VEGF family of receptors. FLT1 is selectively activated by PGF, KDR is selectively activated by VEGFE, and both receptors can also be activated by VEGFA. FLT1 and KDR contain intrinsic tyrosine kinase domains. Panel (**B**). PGE2 family of receptors. All four prostaglandin receptors (PTGER 1–4) can be activated by PGE2. Selective agonists for each receptor include 17-phenyltrinor PGE2 (17-PTP for PTGER1), butaprost (But for PTGER2), sulprostone (Sul for PTGER3), and PGE1 alcohol (PGE1OH for PTGER4). Panel (**A**) adapted from [37]. Panel (**B**) adapted from [38]. Created in BioRender. Yeshua, A. (2026) https://BioRender.com/tnziz1j, accessed on 30 June 2026.

**Figure 2:**
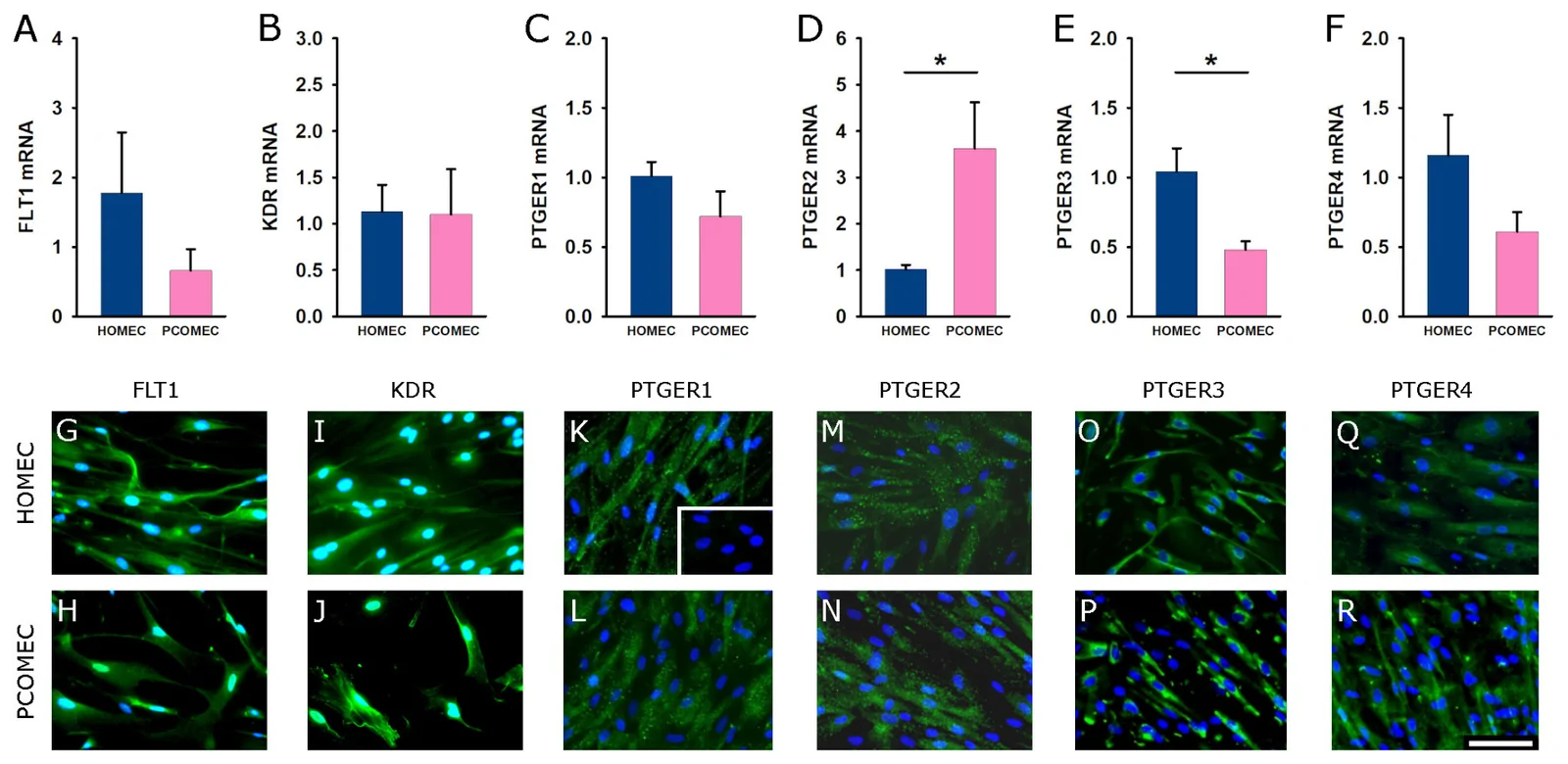
VEGF and PGE2 receptor expression in HOMECs and PMOMECs. mRNA levels for FLT1 (**A**), KDR (**B**), PTGER1 (**C**), PTGER2 (**D**), PTGER3 (**E**), and PTGER4 (**F**) in HOMECs and PMOMECs. For each sample, receptor mRNA was expressed relative to GAPDH mRNA. *N* = 4–6 cell lines/group. PTGER2 and PTGER3 mRNAs are different between HOMECs and PMOMECs by unpaired *t*-test, *p* < 0.05 (as denoted by the asterisk); Panels lacking an asterisk have no significant differences. Immunofluorescent detection of receptor proteins (green) FLT1 (**G**,**H**), KDR (**I**,**J**), PTGER1 (**K**,**L**), PTGER2 (**M**,**N**), PTGER3 (**O**,**P**), and PTGER4 (**Q**,**R**) in HOMECs (**G**,**I**,**K**,**M**,**O**,**Q**) and PMOMECs (**H**,**J**,**L**,**N**,**P**,**R**). Nuclei are counterstained with DAPI (blue). Inset in panel (**K**) shows absence of receptor detection when primary antibodies were omitted. Images in Panels (**G**–**R**) are representative of *n* = 4 HOMEC lines and *n* = 5 PMOMEC lines. All images at same magnification; scale bar in Panel R = 100 µm.

**Figure 3:**
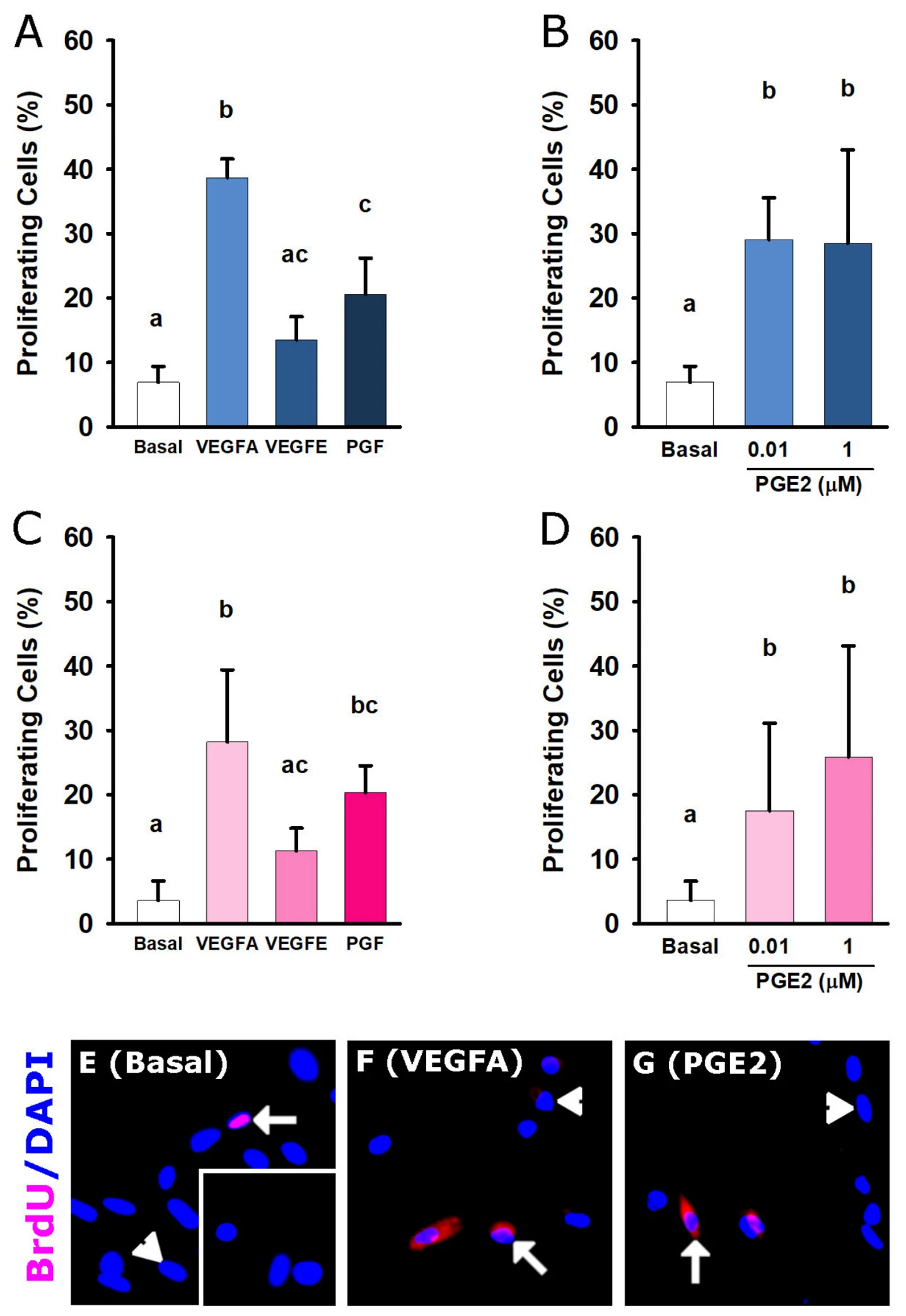
HOMECs and PMOMECs proliferate in response to VEGF receptor agonists and PGE2. Proliferating HOMECs (**A**,**B**) and PMOMECs (**C**,**D**) were identified by BrDU incorporation. Proliferation was quantified after no treatment (basal) or treatment with VEGFA (5 ng/mL), VEGFE (5 ng/mL), PGF (5 ng/mL), or PGE2 (0.01 μM or 1 μM). Data are expressed as the percentage of BrDU+ (proliferating) cell compared with total cells. *N* = 4 cell lines/group. For each panel, groups with no common letter are statistically significantly different (*p* < 0.05, repeated measures ANOVA with Duncan’s post hoc test); panels or groups sharing a letter or lacking letters have no significant differences. Representative images of BrdU detection (pink/red) after no treatment (basal, (**E**)) or treatment with VEGFA (**F**) or 1 µM PGE2 (**G**). Nuclei were counterstained with DAPI (blue). Arrows indicate BrDU detection; arrowheads indicate nuclei lacking BrdU detection. Inset in Panel (**E**) shows absence of BrDU detection when the primary antibody was omitted.

**Figure 4:**
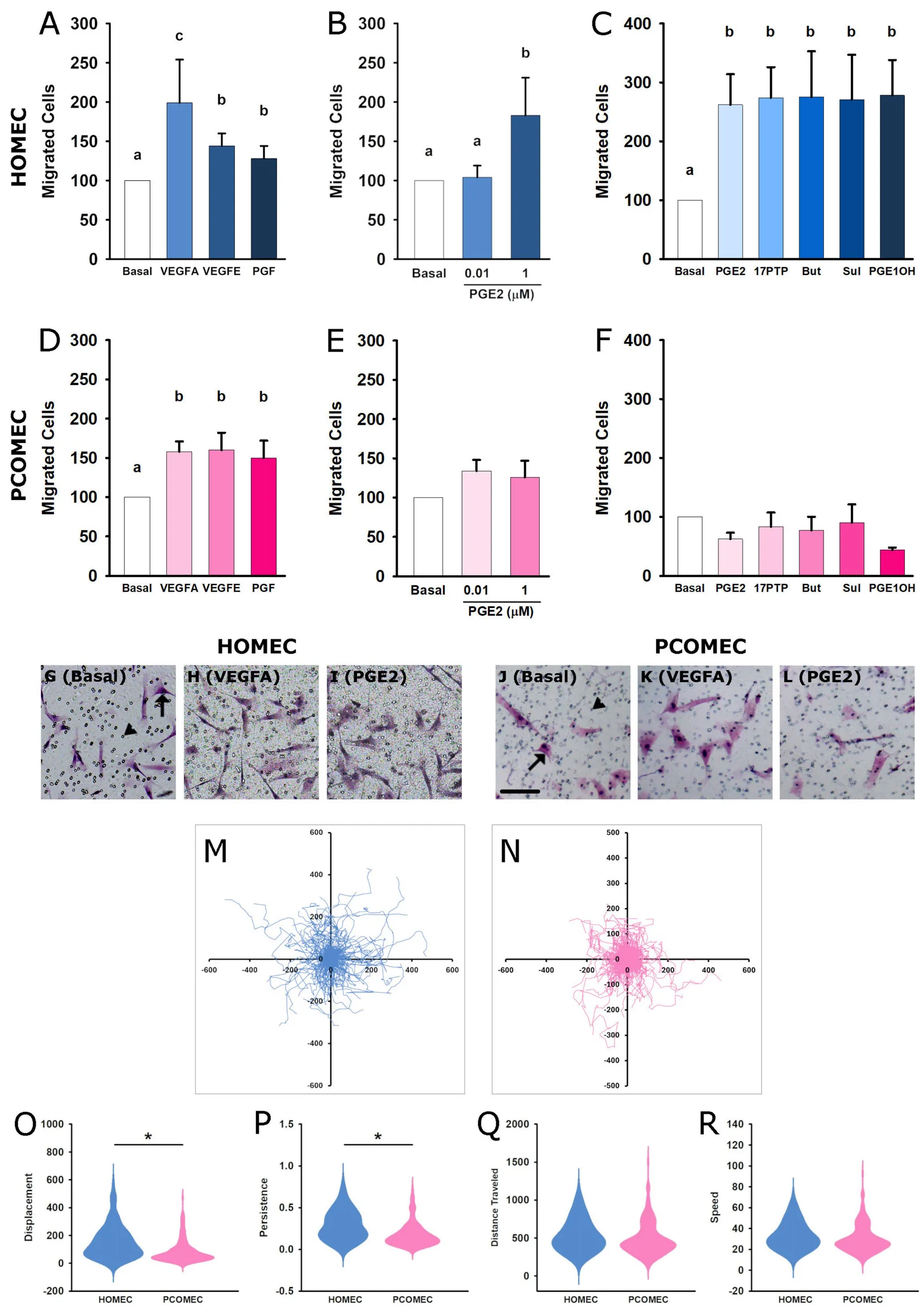
HOMEC and PMOMEC migration in response to VEGF receptor agonists and PGE2 receptor agonists. HOMECs (**A**–**C**) and PMOMECs (**D**–**F**) migrated through a porous membrane in response to no treatment (basal), VEGFA, VEGFE, PGE2 (0.01 µM or 1 µM), 17-phenyltrinor PGE2 (17PTP), butaprost (But), sulprostone (Sul), or PGE1 alcohol (PGE1OH) for 24 h. For each cell line, the number of migrated cells after treatment was normalized to the basal group, which was set equal to 100%. *N* = 4 cell lines/group. For each panel, groups with no common letter are statistically significantly different (*p* < 0.05, repeated measures ANOVA with Duncan’s post hoc test); panels or groups sharing a letter or lacking letters have no significant differences. Images show migrated HOMECs (**G**–**I**) and PMOMECs (**J**–**L**); examples of migrated cells (arrows) and migration pores (arrowheads) are indicated in G and J. All images (**G**–**L**) are shown at the same magnification; scale bar is shown in Panel (**J**) = 100 µm. Spaghetti plots track migration of individual HOMECs (**M**) and PMOMECs (**N**). Original position of each cell is aligned at the center of the grid, and each line tracks the movement of an individual cell over the 16 h migration period. Violin plots show displacement (**O**), persistence (**P**), distance traveled (**Q**), and speed (**R**) of HOMECs and PMOMECs. See text for details. For Panels (**M**–**R**), data represent a total of 120 individual HOMECs (30 cells from each of *n* = 4 HOMEC lines) and 150 individual PMOMECs (30 cells from each of *n* = 5 PMOMEC lines). In Panels (**O**,**P**), HOMEC > PMOMEC by linear mixed effects model with significant main effect of cell type, *p* < 0.05 (as denoted by the asterisk); Panels lacking an asterisk have no significant differences.

## Data Availability

The raw data supporting the conclusions of this article will be made available by the authors on request.

## Supplementary Materials

The following supporting information can be downloaded at: https://www.mdpi.com/article/10.3390/cimb48100979/s1.

## Abbreviations

The following abbreviations are used in this manuscript:

HOMECs	Healthy Ovarian Microvascular Endothelial Cells
PMOMECs	PMOS Ovarian Microvascular Endothelial Cells
OMECs	Ovarian microvascular endothelial cells
PMOS	Polyendocrine metabolic ovarian syndrome
PCOS	Polycystic ovary syndrome
VEGFA	Vascular endothelial growth factor A
VEGFE	Vascular endothelial growth factor E
PGF	Placental Growth Factor
FLT1	Fms-Related Receptor Tyrosine Kinase 1 (VEGFR1)
KDR	Kinase Insert Domain Receptor (VEGFR2)
PTGER	Prostaglandin E Receptor
PGE2	Prostaglandin E2
EGM-2	Endothelial Growth Medium-2
EBM-2	Endothelial Basal Medium-2
DAPI	4′,6-diamidino-2-phenylindole
DMSO	Dimethyl sulfoxide
