# Supplementary material for: Ovarian Microvascular Endothelial Cell Migration Is Dysregulated in PMOS
Source: Curr Issues Mol Biol. 2026 Sep 24;48(10):979. doi: 10.3390/cimb48100979 (PMC13703554; doi:10.3390/cimb48100979)
Supplement: Supplementary file 1 [file cimb-48-00979-s001.zip › Table S1.pdf]

Supplemental Table S1

Primers for qPCR.

Target	Forward & reverse sequence	Annealing temperature	Accession #	Cycles
FLT1	F-ACCTCACTGTTCAAGGAACCTC<br>R-AGCCACACAGGTGCATGTTA	58°C	NM_002019.4	45
KDR	FGAGGGGAACTGAAGACAGGC<br>R-GGGCACCATTCCACCAAAAG	60°C	NM_002253.4	40
PTGER1	F-GGACCCAATGCTGGTGTT<br>R-GATCTGGTTCCAGGAGGCAA	59°C	NM_000955.3	40
PTGER2	F-CCTTGCCTTTCACGATTTTTGC<br>R-ACAACAGAGGACTGAACGCA	60°C	NM_000956.4	40
PTGER3	F-CAACCTTTTCTTCGCCTCTGC<br>R-TTTTCAACATCATTATCAGGAGCG	58°C	NM_198715.3	40
PTGER4	F-GAAAGCTGGCAACTCTGACC<br>R-GCAAGGCTGGGTCTGTAGC	58°C	NM_000958.3	40
GAPDH	F-TTCAACAGCGACACCCACTC<br>R-GCCAATTCGTTGTCATACCAGG	62°C	NM_002046.7	40
